# Prevalence of malaria among febrile patients and assessment of efficacy of artemether-lumefantrine and artesunate-amodiaquine for uncomplicated malaria in Dolisie, Republic of the Congo

**DOI:** 10.1186/s12936-022-04143-4

**Published:** 2022-05-02

**Authors:** Brice Pembet Singana, Prisca Nadine Casimiro, Brunelle Matondo Diassivi, Simon Charles Kobawila, Jean-Mermoz Youndouka, Leonardo K. Basco, Pascal Ringwald, Sébastien Briolant, Mathieu Ndounga

**Affiliations:** 1 Faculté des Sciences et Techniques, Université Marien Ngouabi, BP 69 Brazzaville, Republic of the Congo; 2 Institut National de Recherche en Sciences de la Santé, Brazzaville, Republic of the Congo; 3 Programme National de Lutte Contre le Paludisme, Direction Générale de l’Epidémiologie de la Maladie, Ministère de la Santé et de la Population, Brazzaville, Republic of the Congo; 4 Aix Marseille Univ., IRD, AP-HM, SSA, VITROME, Marseille, France; 5 IHU-Méditerranée Infection, Marseille, France; 6 Global Malaria Programme, World Health Organization, Geneva, Switzerland; 7 Unité de Parasitologie Entomologie, Département de Microbiologie et Maladies Infectieuses, Institut de Recherche Biomédicale des Armées, Marseille, France

**Keywords:** Malaria, *Plasmodium falciparum*, Drug resistance, Artemisinin, Artemisinin-based combination therapy, Dolisie, Republic of the Congo

## Abstract

**Background:**

In the Republic of the Congo, malaria represents a major public health problem affecting all age groups. A regular surveillance of the current efficacy of first-line anti-malarial drugs is required in the face of possible emergence and spread of artemisinin-resistant *Plasmodium falciparum* strains in Africa. The purpose of this study was to determine the prevalence of malaria among febrile patients of all ages and assess the efficacy of artemether-lumefantrine (AL) and artesunate-amodiaquine (ASAQ) in Congolese children.

**Methods:**

Febrile patients of all ages were initially screened for malaria by both rapid diagnostic test (RDT) and microscopy. Patients less than 12 years of age, with parasitaemia ≥ 1000 asexual parasites of *P. falciparum*/µL of blood, without any signs of severity, were enrolled in a therapeutic efficacy study and treated after obtaining their parents' (or legal guardian’s) informed consent in two health centres in Dolisie. The patients were followed for 28 days in accordance with the 2009 World Health Organization standard protocol. If parasitaemia reappeared on or after day 7, the genetic profiles (genes expressing merozoite surface protein-1 [*msp1*], merozoite surface protein-2 [*msp2*], and glutamine-rich protein [*glurp*]) of pre-treatment and post-treatment isolates were compared by nested polymerase chain reaction (PCR) followed by capillary electrophoresis to make a distinction between recrudescence and re-infection. The clinical and parasitological outcome was analysed by the per-protocol method and Kaplan–Meier survival curves.

**Results:**

A total of 994 febrile patients of all ages were screened by RDT and microscopy. Of 994 patients, 323 (32.5%) presented a positive RDT, and 266 (26.8%) were microscopy-positive. Based on microscopy as the reference diagnostic method, the sensitivity and the specificity of the RDT were 98.9 and 91.8%, respectively. The Cohen’s kappa coefficient was 0.86. A total of 121 children aged less than 12 years (61 in AL treatment group and 60 in ASAQ treatment group) were included in therapeutic efficacy study. Before PCR correction, the proportions of adequate clinical and parasitological response were 96.6% for AL and 86.0% for ASAQ in the per-protocol population (*P* < 0.05). The PCR-corrected efficacy rates were 98.2% and 94.2% for AL and ASAQ, respectively (*P* > 0.05). Both treatments were well tolerated.

**Conclusions:**

AL and ASAQ remain highly effective for the first-line treatment of uncomplicated *P. falciparum* malaria in Dolisie. Despite high efficacy of first- and second-line treatment, there is a continuing need to scale up effective malaria preventive interventions and vector control strategies in the country.

*Trial Registration Number:* ACTRN12616001422415.

## Background

In the Republic of the Congo, malaria is a major public health problem. According to the World Health Organization (WHO), 117,837 malaria cases were recorded in the country in 2019 [1]. Malaria is the leading cause of presentation at public health centres and hospitals throughout the country, accounting for approximately one-third of medical consultations [2]. The populations at risk include children aged less than 15 years old (more than 60% of uncomplicated malaria and 70% of severe malaria), followed by pregnant women.

The hope for global malaria eradication was dashed in the 1960s with the emergence of *Plasmodium falciparum* strains resistant to chloroquine in South America and Southeast Asia and resistance of *Anopheles* spp. vectors to insecticides [3–5]. In Africa, chloroquine-resistant *P. falciparum* spread rapidly throughout the continent since the late 1970s [6]. In the Republic of the Congo, chloroquine-resistant *P. falciparum*, first reported in 1986, spread to the entire country during the 1990s [7–9]. Sulfadoxine-pyrimethamine (SP) remained clinically effective to treat uncomplicated malaria until the early 2000s [8, 9], but subsequent clinical studies performed after 2003 showed a rapidly declining efficacy of amodiaquine and sulfadoxine-pyrimethamine monotherapies, as supported by in vitro drug sensitivity assays and molecular markers of resistance [10–13]. The emergence and spread of resistance to these anti-malarial drugs have led to the adoption of artemisinin-based combination therapy (ACT) in most countries, as strongly recommended by the WHO [14]. The Republic of the Congo adopted ACT for the first-line treatment of uncomplicated malaria in 2006. Based on the results of randomized therapeutic efficacy studies conducted in 2004 in southern Republic of the Congo [15], artemether-lumefantrine (AL) and artesunate-amodiaquine (ASAQ) were selected by the Congolese Ministry of Health and Population as first- and second-line drugs for the treatment of uncomplicated malaria, respectively [2].

The WHO recommends a regular surveillance of the therapeutic efficacy of first-line anti-malarial drugs at several sentinel sites in malaria-endemic countries [16]. Since the adoption of ACT in 2006, therapeutic efficacy studies have been conducted in three different sites in the Republic of the Congo: Brazzaville, Kindamba (about 100 km to the northwest of Brazzaville, in Pool province), and Owando (about 420 km [510 km by route], northeast of Brazzaville, in Cuvette province) [15, 17–20]. There are no previous data on ACT efficacy from the western part of the country facing the Atlantic Ocean, where Pointe-Noire, often referred to as the economic capital of the country, is situated. In this context, the aim of the present study was to assess the current efficacy of AL and ASAQ in Dolisie, the third largest city of the Republic of the Congo, after Brazzaville and Pointe-Noire.

## Methods

### Study sites

**Fig. 1:**
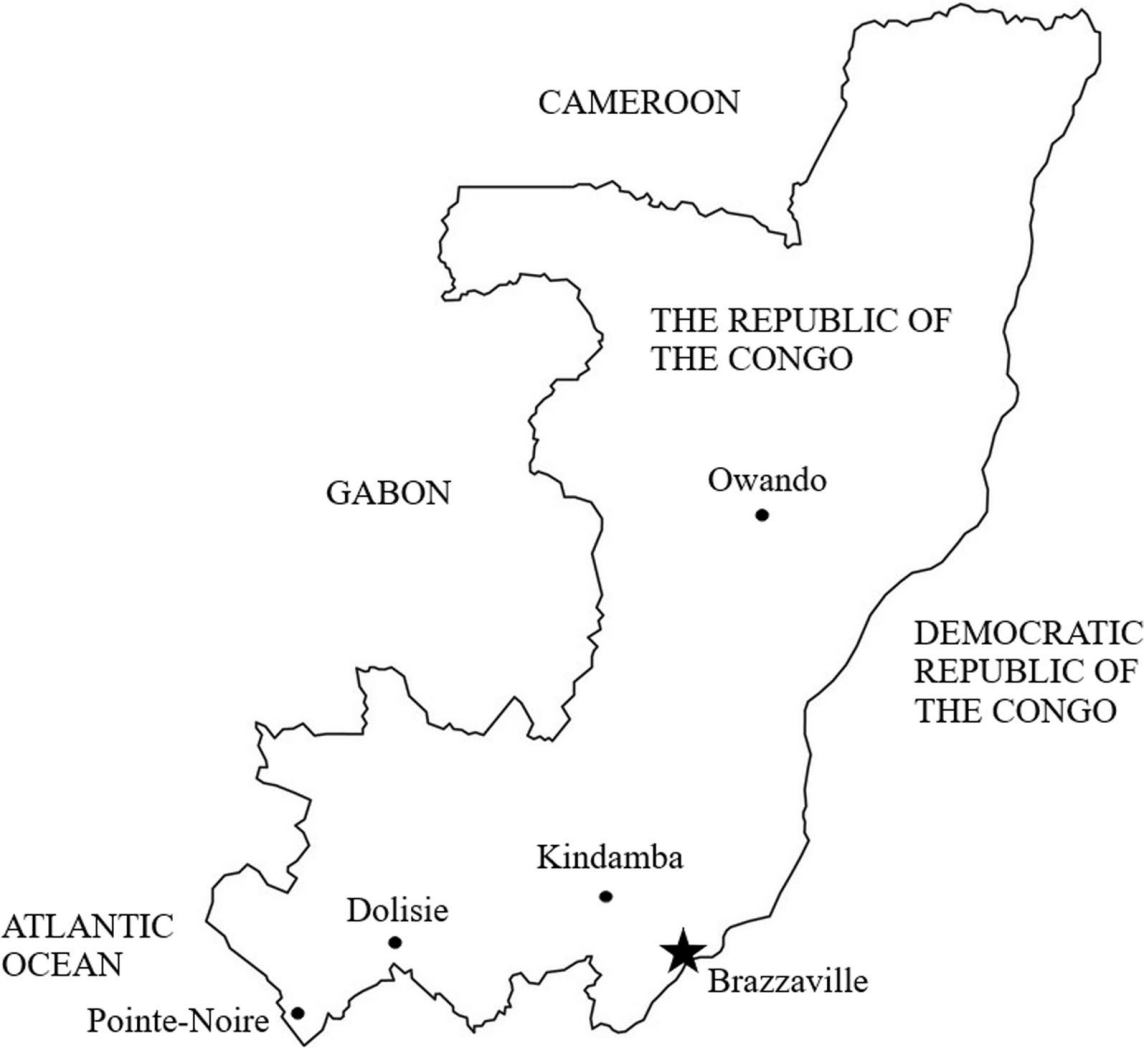
Map of the Republic of the Congo showing the location of Dolisie. Dolisie, the site of the present study, lies along the east–west axis joining Brazzaville (the capital city) and Pointe-Noire (the second largest city) in southern Republic of the Congo. Previous therapeutic efficacy studies (since 2000) on non-artemisinin monotherapies (chloroquine, amodiaquine, SP), non-artemisinin combinations, and ACT were performed in Brazzaville, Owando, Kindamba, and Pointe-Noire

The present study was conducted in Dolisie (formerly called Loubomo; 4°12′29" South and 12°39′3" East), the regional capital of Niari province. Dolisie is located about 350 km west of Brazzaville, the capital city, and 160 km northwest from Pointe-Noire (Fig. 1). The city covers an area of approximately 100 km^2^ [21]. The current (2017) estimated population of Dolisie is 171,773 inhabitants [22]. Agriculture is the main economic activity in Dolisie.

In the Republic of the Congo, two major epidemiological strata can be defined based on geography and climate: (i) tropical forest (60% of the surface area) where the climate is humid and equatorial (annual rainfall > 1 500 mm) in the central and northern regions of the country and (ii) savannah (40% of the surface area) where the climate is humid and tropical (annual rainfall < 1 500 mm) in the south [2, 23]. Malaria transmission is continuous in the equatorial forest in the north, where an estimated entomological inoculation rate (EIR) of > 200 infective bites/person/year occurs, and seasonal (usually June to October) in the savannah in the south, with an estimated EIR < 200 infective bites/person/year [24]. There are no previous data on malaria epidemiology in Dolisie.

The health district of Dolisie is comprised of two hospitals (Reference Hospital and the General Hospital), six integrated public health centres, and numerous private health facilities. The present study was conducted in two public health centres: Dimébéko and Moupépé.

### Study population

Febrile patients consulting one of two health centres were screened for malaria using both rapid diagnostic test (RDT) and microscopy from January 16, 2017 to April 10, 2017. Anthropometric parameters (age, weight, and height), axillary temperature, and results of the RDT (Standard Diagnostic Malaria AG Pf histidine-rich protein-2 (HRP2)/plasmodial lactate dehydrogenase (pLDH); lot 05FDA002A) were recorded on the screening form. The RDT package was a donation from the United Nations Children’s Fund (formerly known as the United Nations International Children’s Emergency Fund; UNICEF) to the Congolese National Malaria Control Programme (NMCP). The test was performed and interpreted in accordance with the manufacturer’s instructions.

After consultation with the physician or nurse, children aged < 12 yrs with a positive RDT result were referred to the laboratory of the health centres for determination of *Plasmodium* species, parasite count based on microscopy, and haematocrit. Few drops of fingerprick capillary blood were obtained to prepare Giemsa-stained thick and thin blood smears, according to the standard WHO-recommended method [25]. Parasite density was determined by counting the number of parasites against 200 leukocytes and expressed as the number of asexual parasites/µL, assuming 8000 leukocytes/µl of blood. Patients with parasitaemia ≥ 1000 asexual parasites/µl of blood and without any sign of danger or concomitant pathology were included after informed consent of their parents or legal guardians [16].

### Treatment and follow-up of patients

A standard dose of AL (20 mg artemether + 120 mg lumefantrine, lot DY1576027; manufactured by Ipca, Mumbai, India) was administered according to body weight, as recommended by the manufacturer: 5–14 kg body weight, 1 tablet per dose (a total of 6 tablets given over 3 days); 15–24 kg body weight, 2 tablets per dose (a total of 12 tablets given over 3 days); 25–34 kg body weight, 3 tablets per dose (a total of 18 tablets given over 3 days); > 35 kg body weight, 4 tablets per dose (a total of 24 tablets given over 3 days). A total of six doses were given. The parents were instructed to administer the second dose at home 8 h after the first dose. The third and fifth doses were administered under supervision during the follow-up in the morning of days 1 and 2. The second, fourth and sixth doses were given to the parents for administration at home during the evening of day 0 (8 h after the first dose), day 1, and day 2. For better absorption, each dose was administered with milk.

Three different batches of ASAQ manufactured by Sanofi Aventis (Casablanca, Morocco) were available for the study: batch 5MA392 for 100 mg artesunate + 270 mg amodiaquine, batch 6MA095 for 50 mg artesunate + 135 mg amodiaquine, and batch 5MA082 for 25 mg artesunate + 67.5 mg amodiaquine. The patients received once daily dose of ASAQ for three days under supervision according to their weight, as recommended by the manufacturer: 4.5– < 9 kg body weight, 1 tablet of ASAQ 25 mg/67.5 mg; 9– < 18 kg body weight, 1 tablet of ASAQ 50 mg/135 mg; 18– < 36 kg body weight, 1 tablet of ASAQ 100 mg/270 mg; ≥ 36 kg body weight, 2 tablets of ASAQ 100 mg/270 mg. ASAQ was administered with mineral water.

The first dose was given at the health centre under supervision. The patients were observed for 30 min after drug intake to rule out vomiting. If a patient vomited during the observational period of 30 min, another complete dose was given, and the patient was observed for another 30 min. If the patient vomited the dose for the second time, the patient was excluded from the study, and parenteral artesunate or artemether was given. All anti-malarials used in this study were provided by the Global Malaria Programme, Geneva, Switzerland.

The follow-up included clinical evaluation and microscopic examination of blood smears on days 1, 2, 3, 7, 14, 21 and 28. The outcome was classified as follows: early therapeutic failure (ETF), late clinical failure (LCF), late parasitological failure (LPF), or adequate clinical and parasitological response (ACPR) [16].

The patients excluded from the study were of four types: lost to follow-up, protocol violation, voluntary withdrawals, and involuntary withdrawals. Since parasitological cure is the primary objective of anti-malarial treatment, any patient presenting with treatment failure (i.e., ETF, LCF, or LPF) received a rescue treatment (if initially treated with AL, ASAQ was administered after AL failure, and vice versa).

All adverse events were noted in the patient's clinical file. Safety was assessed by recording the nature and incidence of adverse events. An adverse event was defined as any unforeseen, unfavourable sign, symptom, syndrome or disease that occurred with the use of a drug, whether or not related to that drug.

### Parasite genotyping

Three additional drops of fingerprick capillary blood (approximately 150 µL) were collected on day 0 when preparing blood smears for microscopic examination and spotted onto Whatman filter paper for parasite genotyping. Fingerprick capillary blood (approximately 150 µL) was also collected and spotted on filter papers on the day of therapeutic failure. Blood spots were dried and stored for parasite genotyping to differentiate between recrudescence (i.e., reappearance of the parasites present on day 0) and new infection. Dried blood spots were sent to Institut Pasteur in Phnom Penh, Cambodia. The genotypic profiles of the parasites at day 0 and day of recurrence were compared to determine whether the recurrent infections were a recrudescence (same strain, i.e. same genotype) or a new infection (different strain, i.e. different genotype), according to the current WHO-recommended algorithm [26]. Briefly, genes expressing merozoite surface protein-1 (*msp1*), merozoite surface protein-2 (*msp2*), and glutamine-rich protein (*glurp*) were amplified by nested polymerase chain reaction (PCR). PCR products were separated by size by capillary electrophoresis. Primary end-point analysis was performed with three polymorphic markers. Recrudescence was defined as recurrent parasitaemia with at least one common allele of each of three markers (3/3) in paired pre-treatment and post-treatment samples.

As an explanatory endpoint, reinfection and recrudescence were also determined by newly proposed two out of three (2/3) algorithm [27]. In this strategy, classification of recurrent failures is based on a consensus result of *msp1* and *msp2* and disparate results are resolved by *glurp*. Such analysis demands concomitant results of at least two markers for classification of reinfection or recrudescence (defined as the presence of at least one common allele in two markers [2/3] in paired pre-treatment and post-treatment samples), compared to three with the standard WHO methodology.

### Statistical analysis

A non-randomized study was performed due to logistic difficulties involved in a randomized design for two artemisinin-based combinations that have different number of doses. The sample size of the patients to be included was based on the assumption of 5% treatment failure rate for each drug. For a confidence level of 95% and 10% precision, a minimum of 120 patients, including 20% to account for loss-to-follow-up and withdrawal during the 28-day follow-up period (i.e., 50 patients + 20% of 50 = 60 patients per treatment group), were recruited and assigned to one of two treatment groups.

The individual patient data recorded on the screening form were entered into a pre-programmed Microsoft Excel^®^ spreadsheet prepared and provided by the WHO Drug Resistance Global Malaria Programme [16]. This file allows two methods of analysis: per-protocol analysis and Kaplan–Meier analysis. The per-protocol method excludes patients lost to follow-up or those who were excluded from analysis and allows comparison of the proportions of ACPR with previous results from other Congolese sites. Kaplan–Meier analysis calculates the probability of the time to treatment failure during the 28-day follow-up period. Survival curves were plotted and analysed using Prism 4.0 software (GraphPad Software, Inc., La Jolla, CA). Survival curves were compared using the logrank test. The percentages of patients with a positive RDT and a positive blood smear were determined by health centre and age groups (< 5 years, 5– < 10 years, 10–14 years, and ≥ 15 years).

Sensitivity, specificity, positive predictive value, negative predictive value were calculated using the exact binomial method. The degree of agreement between microscopy and RDT results was determined using kappa (κ) statistics [28]. The strength of agreement based on the kappa coefficient was graded as follows: < 0, poor agreement; 0–0.20, slight agreement, 0.21–0.40, fair agreement; 0.41–0.60, moderate agreement; 0.61–0.80, substantial agreement; and 0.81–1.00, almost perfect agreement [29].

## Results

### Prevalence of malaria among febrile patients

A total of 994 febrile patients of all ages were screened, 491 at Dimébéko integrated health centre and 503 at Moupépé integrated health centre. One hundred twenty-nine (26.3%; 95% confidence interval [95% CI], 22.4–30.4%) patients presented a positive RDT at Dimébéko health centre and 194 (38.6%; 95% CI, 34.3–43.0%) at Moupépé health centre (*P* < 0.05). One hundred three (21.0%; 95% CI, 17.5–24.9%) and 163 (32.4%; 95% CI, 28.3–36.7%) patients were microscopy positive at Dimébéko and Moupépé health centres, respectively (*P* < 0.05).

### Performance of rapid diagnostic test

The distribution of RDT-positive and microscopy-positive patients according to age groups is presented in Table 1. The performance of the RDT compared to microscopy (reference method) was as follows: sensitivity, 98.9%; specificity, 91.8%; positive predictive value, 81.4%; and negative predictive value, 99.6% (Table 2). The kappa coefficient was 0.86, which corresponds to an “almost perfect agreement” between the two diagnostic methods according to the classification of Landis and Koch [29].

**Table 1:** Malaria screening in Dolisie

Age group	CSI Dimébéko n (%) [95% CI]			CSI Moupépé n (%) [95% CI]			Total n (%) [95% CI]		
	Total screened	RDT positive	TF positive	Total screened	RDT positive	TF positive	Total screened	RDT positive	TF Positive
All patients	491	129 (26.3) [22.4–30.4]	103 (21.0) [17.5–24.8]	503	194 (38.6) [34.3–43.0]	163 (32.4) [28.3–36.7]	994	323 (32.5) [29.6–35.5]	266 (26.8) [24.0–29.6]
< 5 yr	191	27 (14.1) [9.5–19.9]	20 (10.5) [6.5–15.7]	188	38 (20.2) [14.7–26.7]	28 (14.9) [10.1–20.8]	379	65 (17.2) [13.5–21.3]	48 (12.7) [9.5–16.4]
5– < 10 yr	118	44 (37.3) [28.6–46.7]	37 (31.4) [23.1–40.5]	133	72 (54.1) [45.3–62.8]	61 (45.9) [37.2–54.7]	251	116 (46.2) [39.9–52.6]	98 (39.0) [33.0–45.4]
10–14 yr	50	25 (50.0) [35.5–64.5]	19 (38.0) [24.7–52.8]	60	43 (71.7) [58.6–82.5]	40 (66.7) [53.3–78.3]	110	68 (61.8) [52.1–70.9]	59 (53.6) [43.9–63.2]
≥ 15 yr	132	33 (25.0) [17.9–33.3]	27 (20.5) [13.9–28.3]	122	41 (33.6) [25.3–42.7]	34 (27.9) [20.1–36.7]	254	74 (29.1) [23.6–35.1]	61 (24.0) [18.9–29.8]

*n* number of patients, *95% CI* 95% confidence interval, *RDT* rapid diagnostic test, *TF* thick film (microscopy-positive). The proportions of positive RDT and TF in the two health centres were statistically different (*P* < 0.05)

**Table 2:** Performance of the rapid diagnostic test used in the study

Reference method	RDT positive	RDT negative	Total
Microscopy positive	263	3	266
Microscopy negative	60	668	728
Total	323	671	994

The performance of the Standard Diagnostic Malaria AG Pf HRP2/pLDH rapid diagnostic test (RDT) compared to microscopy (reference method) was as follows: sensitivity, 98.9%; specificity, 91.8%; positive predictive value, 81.4%; and negative predictive value, 99.6%. The kappa coefficient was 0.86, which corresponds to an “almost perfect agreement” between the two diagnostic methods, according to the classification proposed by Landis and Koch [29]

### Drug efficacy and safety

**Fig. 2:**
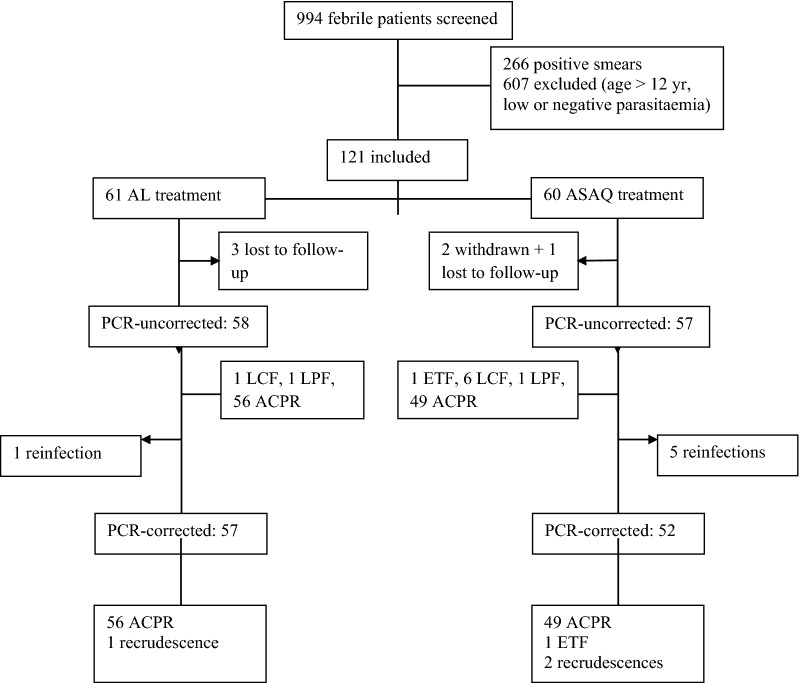
Enrolment and follow-up profile

A total of 121 patients were included, 45 (37.2%) from Dimébéko health centre and 76 (62.8%) from Moupépé health centre (Fig. 2). Sixty-one were assigned to AL treatment group, and 60 to ASAQ treatment group. The characteristics of the patients at inclusion are summarized in Table 3. The mean (± standard deviation, SD; range) age of the patients was 6.8 ± 3.1 (1–12) and 7.6 ± 3.2 (1–12) years for the patients treated with AL and ASAQ, respectively. The geometric mean parasite density was 55,400 and 23,300 asexual parasites/µl for AL and ASAQ treatment groups, respectively. Ten patients had a parasite density > 200,000 asexual parasites/µl but did not present any signs of severe malaria. These patients were included in the study and treated with AL (n = 8) or ASAQ (n = 2).

**Table 3:** Characteristics of malaria-infected Congolese children included in Dolisie

Characteristics	AL	ASAQ
Number of patients	61	60
Mean age (± SD; range) (yr)	6.8 ± 3.1 (1–12)	7.6 ± 3.2 (1–12)
Sex ratio (M/F)	0.9 (29/32)	1.1 (31/29)
Age group		
5–15 yrs < 5 yrs	47 14	46 14
Mean axillary temperature (± SD) (°C)	38.2 ± 1.2	38.0 ± 1.3
Mean weight (range) (kg)	20.7 (9.5–39.0)	23.1 (11.0–45.0)
Geometric mean parasite density (range) (asexual parasites/µl)	55,400 (4,030–490,000)	23,300 (1,060–315,000)
Haematocrit (mean ± SD) (%)	31.1 ± 7.1	32.7 ± 6.9

*SD* standard deviation, *AL* artemether-lumefantrine, *ASAQ* artesunate-amodiaquine

On day 1, 11 of 61 (18.0%) and 2 of 60 (3.3%) patients treated with AL or ASAQ still had fever, respectively. On day 2, none of the patients treated with AL had fever, while one ASAQ-treated patient presented with a low-grade fever. On day 2, three patients in the AL group (118, 93, and 16 asexual parasites/µL of blood) and three patients in the ASAQ group (32, 32 and 100 asexual parasites/µL of blood) still had low parasitaemia. On day 3, all patients were aparasitaemic.

Before PCR correction, the proportions of ACPR were 96.6% (95% CI, 88.3–99.6%) for the AL treatment group and 86.0% (95% CI, 74.6–93.7%) for the ASAQ treatment group (*P* < 0.05) in the per-protocol population (Table 4). After PCR correction, the proportions of ACPR were 98.2% for AL and 94.2% for ASAQ (*P* > 0.05). Of 4 failures, 3 were classified as LCF (1 in the AL group and 2 in the ASAQ group), and 1 patient experienced an ETF after ASAQ treatment. Three children experiencing LCF were treated with an alternative ACT (i.e., if initially treated with AL, ASAQ was administered after AL failure, and vice versa). The patient with ETF was a girl aged 3 years old weighing 12 kg who presented with 2 600 *P. falciparum* asexual parasites/µL and a body temperature of 36 °C on day 0 (with a history of fever less than 48 h before inclusion). The haematocrit on day 0 was 28% (approximately 9.3 g/dL). On day 2, this patient was referred to a tertiary hospital for aggravation of malaria-associated symptoms (day 2 parasite density, 100 asexual parasites/µL). Using the 2/3 algorithm instead of the current WHO-recommended algorithm [26], the PCR-corrected results were similar for AL, whereas one case of reinfection in the ASAQ group was reclassified as recrudescence, ending up with a proportion of ACPR of 92.5%.

**Table 4:** Treatment response of Congolese children to AL and ASAQ in Dolisie

Outcome	Number of patients (%; 95% CI)	
	AL	ASAQ
Number of patients	61	60
PCR‑uncorrected outcome		
Withdrawn or lost to follow up	3	3
Per protocol population	58	57
ETF [95% CI]	0 (0) [0–6.2]	1 (1.8) [0–9.4]
LCF [95% CI]	1 (1.7) [0–9.2]	6 (10.5) [4.0–21.5]
LPF [95% CI]	1 (1.7) [0–9.2]	1 (1.8) [0–9.4]
ACPR [95% CI]	56 (96.6) [88.1–99.6]	49 (86.0) [74.2–93.7]
PCR corrected outcome		
Withdrawn	0	2
Lost to follow up	3	1
Reinfection (censored)	1	5
Per protocol population	57	52
ETF [95% CI]	0 (0) [0–6.3]	1 (1.9) [0–10.3]
LCF [95% CI]	1 (1.8) [0–9.4]	2 (3.8) [0.5–13.2]
LPF [95% CI]	0 (0) [0–6.3]	0 (0) [0–6.8]
ACPR [95% CI]	56 (98.2) [90.6–100]	49 (94.2) [84.1–98.8]

*95% CI* 95% confidence interval, *ACPR* adequate clinical and parasitological response, *AL* artemether-lumefantrine; *ASAQ* artesunate-amodiaquine, *ETF* early therapeutic failure, *LCF* late clinical failure, *LPF* late parasitological failure, *PCR* polymerase chain reaction

**Fig. 3:**
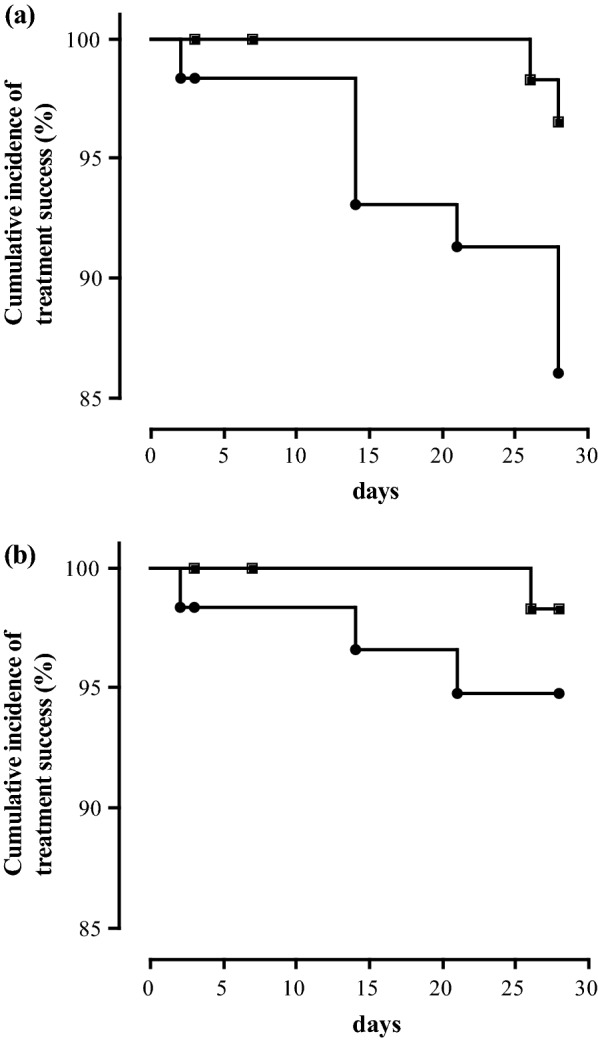
Kaplan-Meier survival curves. **a** Survival curves of patients treated with artemether-lumefantrine (black squares) or artesunate-amodiaquine (black circles) before PCR correction. **b** Survival curves of patients treated with artemether-lumefantrine (black squares) or artesunate-amodiaquine (black circles) after PCR correction

The PCR-uncorrected cumulative incidence curve for therapeutic success of patients treated with AL showed a cumulative incidence of 100% until day 25. After the occurrence of two failures (1 on day 26 and another on day 28), the cumulative incidence at the end of the 28-day follow-up was 96.6% (95% CI, 86.9–99.1%) (Fig. 3a). The uncorrected cumulative incidence after ASAQ treatment on day 28 was 86.0% (95% CI, 74.0–92.8%). Failures occurred on day 2 (1 ETF), day 14 (3 LCF), day 21 (1 LCF), and day 28 (2 LCF + 1 LPF). The comparison of the survival curves by logrank test showed a statistically significant difference between the efficacy of AL and ASAQ before PCR correction (chi-square = 4.10; *P* = 0.043). The Kaplan–Meier curves after PCR correction showed a cumulative incidence of treatment success of 98.3% (95% CI, 88.4–99.8%) on day 28 with AL (Fig. 3b). With ASAQ, the cumulative incidence was 94.7% (95% CI, 84.5–98.3%) on day 28. Although a slightly higher efficacy was observed with AL, compared to ASAQ (98.3% vs 94.2%), the logrank test did not show any statistically significant difference (chi-square, 1.131, *P* = 0.288) between the two curves after PCR correction.

*Plasmodium falciparum* gametocytes were found more frequently (*P* < 0.05) in the ASAQ treatment group than in the AL group during the first 14 days after treatment (Table 5). Among patients presenting with gametocytes before treatment and those with gametocytaemia during the first 3 days after treatment, none had detectable gametocytes by day 28.

**Table 5:** Proportions of Congolese children with *Plasmodium falciparum* gametocytaemia before and after treatment with artemether-lumefantrine and artesunate-amodiaquine in Dolisie

Days after treatment	Number of patients with gametocytes/number of patients in the per protocol population (%)	
	Artemether-lumefantrine	Artesunate-amodiaquine
Day 0	5/61 (8.2)	8/60 (13.3)
Day 2	7/61 (11.5)	14/60 (23.3)
Day 3	6/60 (10.0)	12/55 (21.8)
Day 7	3/58 (5.2)	11/56 (19.6)
Day 14	2/58 (3.4)	6/55 (10.9)
Day 21	0/58 (0)	1/50 (2.0)
Day 28	0/58 (0)	0/50 (0)

Asthenia, abdominal pain, vomiting, loss of appetite, and facial oedema (n = 2) were reported by AL-treated patients (Table 6). Patients in the ASAQ group reported abdominal pain, vomiting, asthenia, and loss of appetite more frequently (*P* < 0.05) than those in the AL group. In addition, one ASAQ-treated patient complained of headache, and another ASAQ-treated patient reported pruritus accompanied by rash. None of the children reported nausea, diarrhoea, dizziness, or icterus.

**Table 6:** Adverse events reported by children treated with artemether-lumefantrine or artesunate-amodiaquine

Adverse effects	Number of patients									
	Artemether-lumefantrine					Artesunate-amodiaquine				
	D1	D2	D3	D7	Total	D1	D2	D3	D7	Total
Number of patients	61	61	61	60	243	60	60	57	56	233
Asthenia	0	2	1	0	3	13	12	7	0	32
Loss of appetite	0	1	0	0	1	6	7	0	0	13
Vomiting	1	2	0	0	3	8	5	1	0	14
Abdominal pain	0	1	1	1	3	3	3	1	0	7
Headache	0	0	0	0	0	1	1	0	0	2
Pruritus	0	0	0	0	0	1	0	0	0	1
Facial oedema	0	1	2	0	3	0	0	0	0	6
Total	1	7	4	1	13	32	28	9	0	75

The numbers of adverse events are reported on each follow-up day until day 7. If the same child reported the same adverse effects on different days, the total number of cumulative adverse events was reported. None of the children complained of nausea, dizziness, diarrhoea, or icterus. *D* day after inclusion in the study (day 0 is the first day of inclusion)

## Discussion

The prevalence of laboratory-confirmed malaria was relatively high (266 of 994 by microscopy, 26.8%; 323 of 994, 32.5% by RDT) among febrile patients presenting spontaneously at the health centres in Dolisie, as expected. Several studies conducted in the 2010s in Owando, Pointe-Noire, and Brazzaville also showed that about 12–36% of febrile children screened for malaria by microscopy, RDT, and/or PCR were positive for malaria, depending on the season and study site [20, 30–34]. In earlier studies conducted in the 2000s, similar or higher proportions of microscopy-confirmed malaria had been observed in Brazzaville (23.8% in a health centre in an urban site; 44.7% in a health centre situated in a semi-rural area) [35]. A comparison of data on the proportion of laboratory-confirmed malaria among febrile Congolese children between prevalence studies conducted in the 2000s and those performed in the 2010s suggests that malaria prevalence may be decreasing, at least in Brazzaville. This apparent diminution of malaria cases based on individual studies does not appear to be age-dependent. In Dolisie, a lower proportion of febrile children aged less than 5 years old (12.7% by microscopy) were malaria-positive, as compared to older children. Similar observations were reported in Brazzaville in studies conducted in 2003–2006 and 2015–2016 [34, 35]. On the national level, there is limited evidence of decrease in the number of malaria cases in the Republic of the Congo between 2000 and 2009 [36]. However, more recent longitudinal data from the Republic of the Congo suggest that, over the past two decades, the estimated number of malaria cases decreased considerably if population increase is taken into consideration (mean estimated annual number of malaria cases/population: 1,107,773 malaria cases/3,127,420 [35.4%] in 2000 vs 1,241,940 malaria cases/5,380,504 [23.1%] in 2019) [1]. This latter observation is probably related to an improved access of the population to diagnosis and treatment provided in different health structures, the widespread use of ACT for laboratory-confirmed malaria cases, implementation of intermittent preventive treatment in pregnant women, mass distribution of insecticide-impregnated bed nets, and increasing awareness and knowledge of the population concerning malaria [1, 24]. Although these data are encouraging and tend to support the effectiveness of current intervention strategy to control malaria, much more effort will be needed for the Republic of the Congo to enter into the pre-elimination phase.

Since 2004, 13 therapeutic efficacy studies (both published and unpublished studies; 8 for AL and 5 for ASAQ) have been conducted in the Republic of the Congo [37, 38]. The earlier studies showed that AL and ASAQ are highly effective (> 97.5% ACPR) in Brazzaville, Kindamba, and Owando [15, 17–20]. The present work is the first study on therapeutic efficacy and evaluation of the prevalence of uncomplicated malaria among febrile children performed in Dolisie, 11 years after the change of national drug policy for the treatment of uncomplicated malaria. AL and ASAQ remained highly effective and well tolerated for the treatment of uncomplicated *P. falciparum* malaria in children in Dolisie, with PCR-corrected ACPR well above the 90% threshold set by the WHO for an effective first-line ACT [16]. Reinfections occurred more often (i.e., 8 of 51; 16%) after treatment with ASAQ than with AL. The study performed in Owando also showed a slightly higher rate of re-infection after ASAQ (4/55, 7.3%), compared to AL (3/52, 5.8%) [20]. Although these results are surprising considering the longer post-treatment prophylactic effect of ASAQ, which usually results in lower reinfection rate after ASAQ treatment compared with AL, higher reinfection rates with ASAQ were reported by several authors [39–41]. The elimination half-lives of lumefantrine and amodiaquine were hypothesized to be the underlying explanation by some authors [42], i.e. about 3–5 days for lumefantrine and approximatively 10–14 days for desethylamodiaquine (biologically active metabolite of amodiaquine) [43, 44]. No obvious reasons, including the intensity of malaria transmission, age, or parasite genotype, can explain these unusual results [45]. The two studies were conducted sequentially one month apart, mid-January to the end of February and mid-February to mid-April, including the follow-up period of 28 days, for AL and ASAQ, respectively. Age was similar in both groups, and geometric mean parasite density was higher in the AL group compared to the ASAQ group. It cannot be excluded that parasites in the Republic of the Congo start to show reduced susceptibility to amodiaquine, which would lead at this early stage to reduced prophylactic effect and increased rate of reinfection. Unfortunately, data on molecular markers for anti-malarial drug resistance are missing, limiting interpretation of the clinical outcome.

The present study has some limitations. Assignment to AL and ASAQ treatment arms was not randomized, primarily due to the complexity of logistics and organization of a study involving two therapeutic regimens that differ in the total number of doses. The administration of second, fourth, and sixth AL doses was not supervised because supervision would have required either the patient to return to the health centre at night or one of the research team members to make a home visit. However, despite unsupervised administration of 3 of 6 AL doses, AL was highly effective. The rate of reinfection was relatively high after ASAQ treatment in the present study, but true recrudescence was rare, and the differences of PCR-corrected ACPR rates and survival curves of AL and ASAQ were not statistically significant. The results obtained in the present study confirm those of earlier studies conducted in the Republic of the Congo [15, 17–20]. To date, the lowest proportion of PCR-corrected ACPR obtained in a therapeutic efficacy study carried out in the Republic of the Congo was 94.4% for ASAQ in 2005 in Brazzaville [18]. The high efficacy and good tolerance of AL and ASAQ are in agreement with those reported from other neighbouring Central African countries [46–52]. In the present study, mature *P. falciparum* gametocytes present before treatment and those that were detected during the first 7 days after treatment were cleared by day 21 or day 28 with both AL and ASAQ, as shown in previous studies [53, 54].

A clinical study conducted in Brazzaville and Pointe-Noire in 1999–2001 suggested a high level of chloroquine resistance and moderate resistance to antifolates, as supported by mutations in molecular markers of resistance to these drugs [9, 12]. An in vitro drug sensitivity study also confirmed the high prevalence of chloroquine- and antifolate-resistant *P. falciparum* isolates collected in Pointe-Noire in 2005–2006 [13]. In that study, amodiaquine, dihydroartemisinin, and lumefantrine were highly active in vitro against multidrug-resistant *P. falciparum*. More recent analysis of *P. falciparum kelch 13* propeller gene, a molecular marker of resistance to artemisinins [55], did not detect any mutation associated with resistance in isolates in Brazzaville, Pointe-Noire, and in northern region of the country [32, 34, 56]. Available data from in vitro drug sensitivity assays, molecular analysis of drug resistance markers, and therapeutic efficacy studies on AL and ASAQ indicate that at present these two artemisinin-based combinations are reliable first-line drugs to treat uncomplicated malaria in the Republic of the Congo. Nonetheless, because partial resistance to artemisinin had become a threatening reality in Southeast Asia, Africa may soon be concerned [57]. Recently, independent emergence of artemisinin partial resistance has been reported in Rwanda and Uganda [58, 59]. ACT failures were reported after AL treatment in the absence of artemisinin partial resistance, but these failures could not be attributed to lumefantrine resistance which has not yet been confirmed in Africa [60]. Therefore, a close surveillance with all available means and tools is required for early detection of the emergence of malaria parasite expressing artemisinin partial resistance or partner drug resistance associated with a rapid response to counter the spread of these parasites in the African continent.

## Conclusions

The results of this study demonstrate that AL and ASAQ remain effective for the first-line treatment of uncomplicated *P. falciparum* malaria in Dolisie. However, continued monitoring of these two artemisinin-based combinations is necessary. Despite high efficacy of both combinations observed in the present study, other complementary interventions, including various preventive strategies (intermittent preventive treatment in pregnancy and in infants, seasonal malaria chemoprevention) and various vector control strategies (effective use of long-lasting insecticide-treated nets, residual insecticide spraying) should be implemented throughout the country.

## Data Availability

The datasets generated and analysed during the current study are available in the repository of Global Malaria Programme, World Health Organization, Geneva, Switzerland.

## Abbreviations

ACPR: Adequate clinical and parasitological response
ACT: Artemisinin-based combination therapy
AL: Artemether-lumefantrine
ASAQ: Artesunate-amodiaquine
CERSSA: Ethics committee for research in health sciences (Comité d’Ethique de la Recherche en Sciences de la Santé)
EIR: Entomological inoculation rate
ETF: Early therapeutic failure
*glurp*: Gene expressing glutamine-rich protein
HRP-2: Histidine-rich protein-2
LCF: Late clinical failure
LPF: Late parasitological failure
*msp1*: Gene expressing merozoite surface protein-1
*msp2*: Gene expressing merozoite surface protein-2
NMCP: National Malaria Control Programme
PCR: Polymerase chain reaction
pLDH: Plasmodial lactate dehydrogenase
RDT: Rapid diagnostic test
SP: Sulfadoxine-pyrimethamine
UNICEF: United Nations International Children’s Emergency Fund (now called United Nations Children’s Fund)
WHO: World Health Organization
